# Effects of sternocleidomastoid muscle and suboccipital muscle soft tissue release on muscle hardness and pressure pain of the sternocleidomastoid muscle and upper trapezius muscle in smartphone users with latent trigger points

**DOI:** 10.1097/MD.0000000000012133

**Published:** 2018-09-07

**Authors:** Seong-Joong Kim, Jung-Hoon Lee

**Affiliations:** a Department of Biomedical Health Science, Graduate School, Dong-Eui University; b Department of Rehabilitation center, Hanyang Rheumatis Hospital, Yeonje-gu; c Department of Physical Therapy, College of Nursing, Healthcare Sciences and Human Ecology, Dong-Eui University, Busan, Republic of Korea.

**Keywords:** Muscle hardness, pressure pain threshold, SCM muscle, soft tissue release, suboccipital muscle, upper trapezius muscle

## Abstract

Few studies have been performed regarding the reduction of pain in the upper trapezius (UT) muscle by applying interventions to the sternocleidomastoid (SCM) muscle, which is innervated by the same nerves.

The purpose of this study was to investigate the effects of soft tissue release intervention on the SCM and suboccipital muscles with regard to muscle hardness and pressure pain threshold (PPT) of the SCM and UT muscles in smartphone users with latent myofascial trigger points (MTrPs) in the UT muscle.

Seventeen smartphone users (5 men and 12 women) with latent MTrPs in the UT muscle participated in the study. This study used a single blinding, cross-over design, wherein sternocleidomastoid soft tissue release (SSTR) and suboccipital release (SR) were applied on the subjects in random order one week apart. Muscle hardness and the PPT of the SCM and UT muscles were assessed before and after the intervention.

After SSTR was applied, the SCM and UT muscles showed a significant decrease in muscle hardness and a significant increase in PPT. After SR was applied, the UT muscle showed a significant decrease in muscle hardness and a significant increase in PPT. When comparing the amount of change between the SSTR and SR interventions, significant differences were found for SCM muscle hardness and PPT of the UT muscle in the SSTR intervention, compared with the SR intervention.

Therefore, we suggest that, to reduce pain in the UT muscle, it may be useful to apply intervention directly to the UT muscle, as well as to the SCM muscle, which is innervated by the same nerve.

## 1 Introduction

Prolonged smartphone use causes continued mechanical stress on the tendons, muscles, and surrounding tissues, and furthermore, maintaining the same posture can also cause musculoskeletal disorders.^[1,2]^ A study by Straker et al^[3]^ reported that using a small video terminal with a smaller screen than a regular desktop computer caused an increase in the activities of the muscles surrounding the neck and shoulders, whereas a study by Park et al^[4]^ reported that prolonged smartphone use induced fatigue in the cervical erector spinae and upper trapezius (UT) muscles. Lee et al^[5]^ also reported that smartphone use increased pain and muscle fatigue in the UT muscle.

The UT and sternocleidomastoid (SCM) muscles are often involved in work-related musculoskeletal disorders (WMSDs) of the upper arms.^[6]^ Deformation of anatomical structures caused by musculoskeletal disorders may compress the nerves,^[7]^ and increased nerve compression can compress the capillaries within the nerves to cause changes in hemodynamics, whereas chronic compression may cause inflammation, fibrosis, and demyelination, which may ultimately lead to axonal loss.^[8]^ Clinical manifestations include sensory disturbance, motor dysfunction, and pain,^[9]^ and such symptoms are referred to as nerve entrapment syndrome. The signs of pain associated with nerve entrapment include severe local pain as well as neuropathic pain, such as paresthesia, tingling, and dysesthesias originating from the entrapped nerve.^[10]^

The spinal accessory nerve runs underneath the SCM muscle or directly passes through the muscle belly, and thus, when myofascial trigger points (MTrPs) are created from a microinjury compress and excite the nerve, pain may be caused by ischemia in the UT muscle that is innervated.^[11]^ Direct interventions for MTrPs of the UT muscle include active release techniques, muscle energy techniques,^[12]^ positional release therapy,^[13]^ and ischemic compression.^[14]^ As shown, to reduce UT muscle pain, it is not only necessary to apply direct intervention to the UT muscle, but also to the SCM muscle and surrounding tissues innervated by the same nerve. However, studies on this topic are still lacking. Accordingly, this study aimed to investigate the effects of the sternocleidomastoid soft tissue release (SSTR) and suboccipital release (SR) on muscle hardness and the pressure pain threshold (PPT) of the SCM and UT muscles in subjects with latent MTrPs in the UT muscle because of prolonged smartphone use.

## 2 Methods

### 2.1 Subjects

A calculation of the sample size using G-Power 3.1 (University of Dusseldorf, Dusseldorf, Germany) for the independent *t* test with a significance level of 0.05, statistical power of 0.80, and effect size of 0.8, estimated the required sample size to be 15. Considering potential drop-out, a total of 17 people (5 men and 12 women; age 20–29 years) were recruited who had MTrP in the UT muscle because of at least 3 hours per day of smartphone use and who consented to participate in the study, which was conducted at Dong-eui University, South Korea. The subject selection criteria were as follows: those with a palpable taut band in the UT muscle^[15]^; presence of a hypersensitive tender spot in the taut band^[15]^; local twitch response provoked by the snapping palpation of the taut band^[15]^; reproduction of the typical referred pain pattern of the MTrPs in response to compression^[15]^; those with pressure pain within 2.5 kg/cm^2^ in the PPT measurement on the taut band in the UT muscle and having at least 1 MTrP ^[13]^; those who use a smartphone for at least 3 hours a day^[4]^; those with no orthopedic or neurological injury 6 months before this study and no history of surgery; and those with no history of taking any medication in the past 3 months. Sociodemographic characteristics of the subjects are shown in Table 1. This study received the approval from the Institutional Review Board at Dong-eui University (DIRB-201707-HR-R-025–01).

**Table 1:**
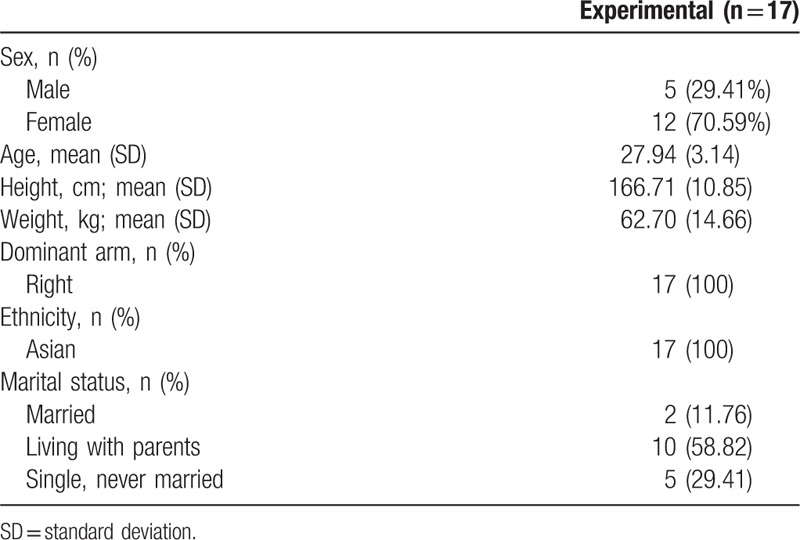
Sociodemographic characteristics of subjects.

### 2.2 Study design

**Figure 1:**
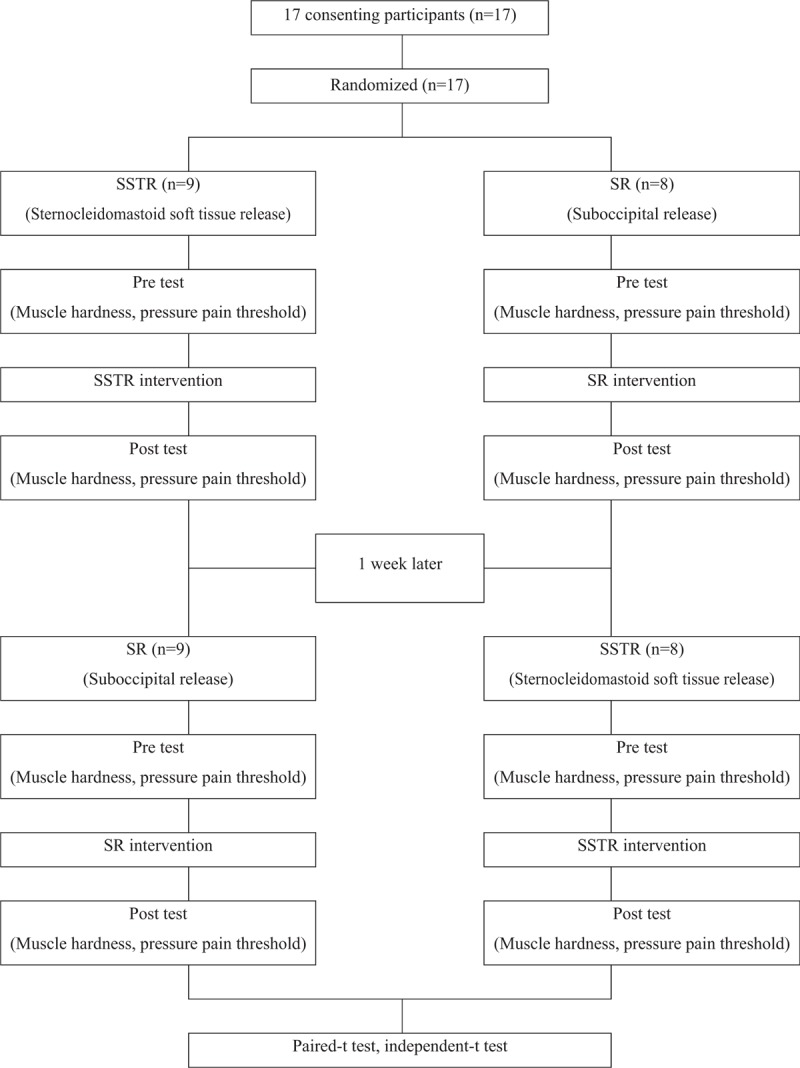
Flow diagram of the study.

This study used single-blinding, cross-over design, wherein SSTR and SR were administered to the subjects in random order with 1 week apart. To investigate the effects of intervention on the muscle hardness and pressure pain of the SCM and UT muscles, muscle hardness and PPT in the SCM and UT muscles were measured before and after the intervention. Figure 1 shows the flow chart illustrating the methods and design used in this study.

### 2.3 Soft tissue release intervention methods

#### 2.3.1 SSTR

**Figure 2:**
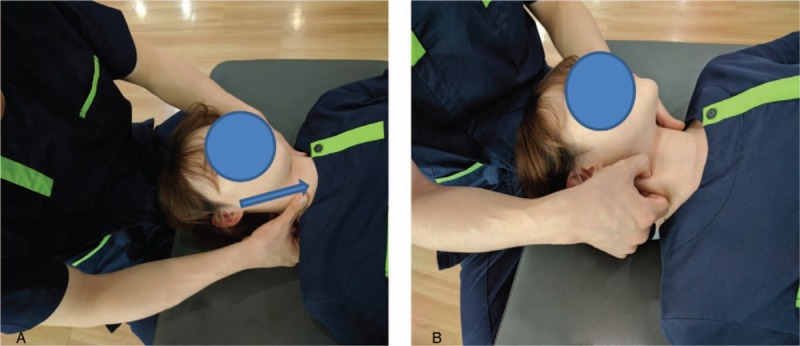
Sternocleidomastoid soft tissue release.

The subject lays in the supine position with both arms straight at their sides, while maintaining external rotation of both shoulders and supination of the lower arms and hands.^[13]^ The SSTR was performed by a physical therapist with at least 8 years of clinical experience. For the SSTR, 5 minutes of stripping and pincer compression techniques were used. The stripping technique involved the therapist placing one hand on top of the subject's head and turning the head in the direction opposite to the intervention area. Then, the therapist placed the tip of the thumb or finger of the other hand on the mastoid process where the SCM muscle is attached. While forcefully pressing down on the SCM muscle, the therapist's thumb was moved slowly to the sternum. During this time, if the point of pressure pain was found, then a constant pressure was maintained until release was achieved. The process from the mastoid process to the sternum, as mentioned above, was repeated (Fig. 2A).^[16]^

The pincer compression technique involved the therapist placing one hand on top of the head and turning the head in the direction opposite to the intervention area. Then, the therapist used the sides of the thumb and index finger on the other hand to hold the area close to the mastoid process of the SCM muscle. If the taut area or area with pressure pain was palpable, pressure was maintained until release was achieved. The fingers were slowly moved downward, repeating this until nearing the manubrium of the sternum (Fig. 2B).^[16]^

#### 2.3.2 SR

**Figure 3:**
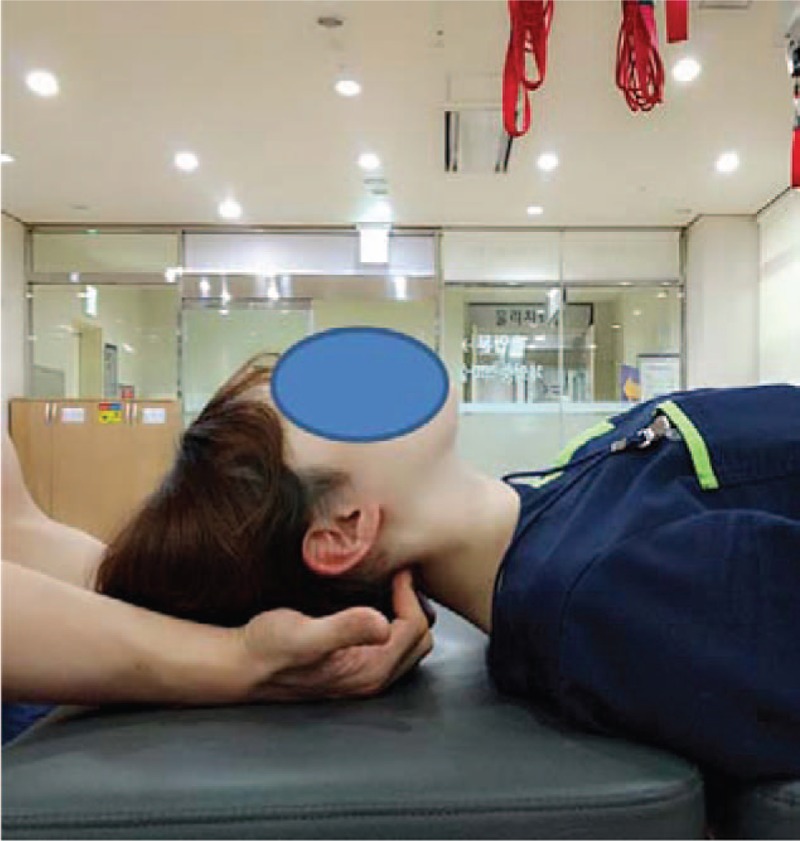
Suboccipital release.

The subject lays in the supine position with both arms straight at their sides, while maintaining external rotation of both shoulders and supination of the lower arms and hands.^[13]^ With the therapist sitting in the direction of the subject's head, the subject's head was placed on top of both of the therapist's hands and the tips of the fingers, excepts the thumbs, were used to hold up the suboccipital muscle, maintaining pressure between the fingers and the suboccipital muscle for 5 minutes while applying a slight traction in the head direction. The SR technique was applied within the range where the subject did not feel any pain (Fig. 3).^[17,18]^

### 2.4 Measurement tools

#### 2.4.1 Neutone

For immediate changes in muscle hardness before and after the SSTR and SR interventions, Neutone (TDM-N1, Tryall, Japan), a muscle hardness tester, was used. Neutone, which can be calibrated by using the formula N = 0.0258 × repulsion force value + 0.4, is a portable device that can measure muscle hardness over a range of 1 to 100 repulsive force values by slowly pressing the target muscle vertically in a relaxed posture.^[19]^ The intra-examiner reliability of Neutone ranged from 0.94 to 0.98.^[20]^

For muscle hardness measurement using Neutone, the subject sat comfortably on a chair with a backrest with both hands placed on top of the knees, while facing forward.^[13]^ The muscle hardness of the SCM muscle was measured from the halfway point between the mastoid process and the manubrium of the sternum, whereas the muscle hardness of the UT muscle was measured from the midpoint of the line connecting the tip of the acromion and the cervical vertebrae 7 (C7) spinous process.^[13]^ The measurement sensors were placed vertically on the skin surface of the SCM and UT muscles. For Neutone data analysis, the mean value (N) of 3 repeated measurements taken 30 seconds apart was used.

#### 2.4.2 Pressure pain algometer

For immediate changes in PPT before and after the SSTR and SR interventions, a pressure pain algometer (Baseline) was used. The pressure pain algometer is a portable instrument for measuring the PPT of a specific muscle; its pressure gauge can be calibrated in kilograms per square centimeter with a 1-cm diameter rubber tip.^[21]^ The intra-examiner correlation coefficients of the PPT, when using the pressure pain algometer, ranged from 0.64 to 0.92.^[21]^

For PPT measurements using the pressure pain algometer, the subject sat comfortably on a chair with backrest with both hands placed on top of the knees, while facing forward.^[13]^ The PPT of the SCM muscle was measured from the halfway point between the mastoid process and the manubrium of the sternum, while the PPT of the UT muscle was measured from the midpoint of the line connecting the tip of the acromion and the C7 spinous process.^[13]^ A metal rod was placed vertically on the skin surface of the measurement area of the SCM and UT muscles, and the subject was instructed to immediately notify the examiner when the pressure applied caused pain rather than just pressure, at which time the pressure was shut off. For data analysis, the mean value (kilograms per square centimeter) of 3 repeated measurements taken 30 seconds apart was used.^[14]^

### 2.5 Analysis method

The study used paired *t* tests to compare the muscle hardness and PPT of the SCM and UT muscles before and after the SSTR and SR interventions. Independent *t* tests were used to compare the amount of change in muscle hardness and PPT of the SCM and UT muscles between the SSTR and SR interventions. Statistical data analysis was performed using SPSS version 18.0 (Version 18.0 for Window, IBM, IL), with statistical significance set at .05.

## 3 Results

### 3.1 Changes in muscle hardness and PPT of the SCM muscle

After SSTR was applied, the SCM muscle showed a significant decrease in muscle hardness and a significant increase in PPT (*P* < .05). After SR was applied, the SCM muscle did not show significant differences in muscle hardness or PPT (*P* *>* .05) (Table 2).

**Table 2:**
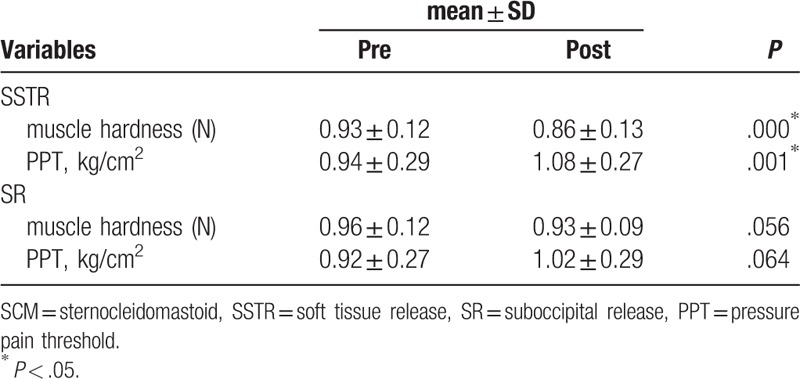
Changes in muscle hardness and pressure pain threshold in the SCM muscle after SSTR and SR interventions.

In the comparison of the amount of change before and after the SSTR and SR interventions, the SCM muscle showed a significant difference in muscle hardness (*P* < .05), but no significant difference in PPT (*P* > .05) (Table 3).

**Table 3:**
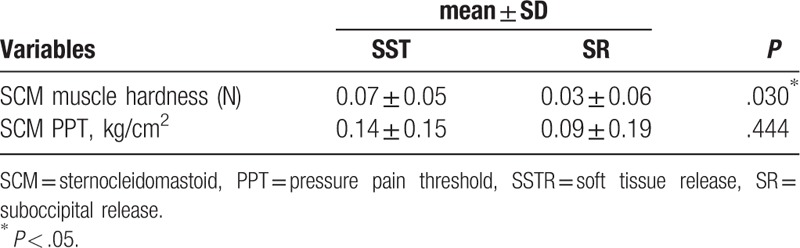
Comparison of the changes in muscle hardness and pressure pain threshold in the SCM muscle in the 2 groups before and after intervention.

### 3.2 Changes in muscle hardness and PPT of the UT muscle

After SSTR was applied, the UT muscle showed a significant decrease in muscle hardness and a significant increase in PPT (*P* < .05). After SR was applied, the UT muscle showed a significant decrease in muscle hardness and a significant increase in PPT (*P* < .05) (Table 4).

**Table 4:**
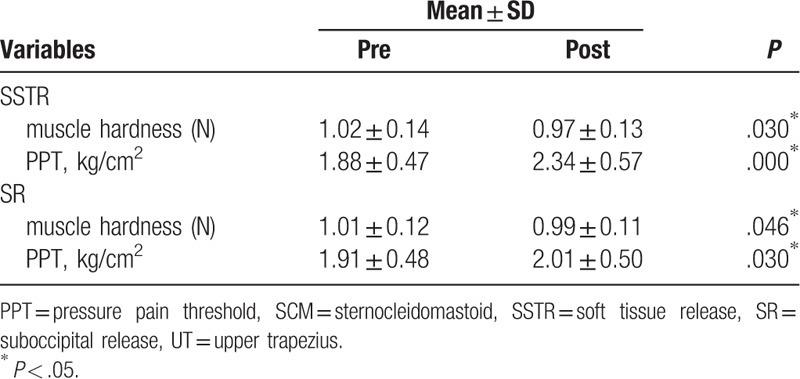
Changes in muscle hardness and pressure pain threshold in the UT after SSTR and SR interventions.

In the comparison of amount of change before and after the SSTR and SR interventions, the UT muscle showed a significant difference in PPT (*P* < .05), but no significant difference in muscle hardness (*p* > .05) (Table 5).

**Table 5:**
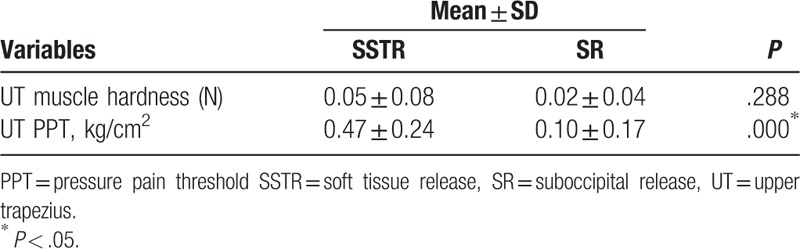
Comparison of the changes in muscle hardness and pressure pain threshold in the UT in the two groups before and after intervention.

## 4 Discussion

The study results showed that after SSTR was applied, muscle hardness decreased in the SCM and UT muscles, while after SR was applied, muscle hardness decreased only in the UT muscle. When comparing the amount of change between the SSTR and SR interventions, the SSTR intervention showed a significant difference in SCM muscle hardness, compared with the SR intervention.

Muscle hardness is defined as the resistance of muscles against pressure applied vertically to the sarcomere.^[22,23]^ When ischemic compression is applied to the MTrP, the vertical width of the sarcomere becomes narrower and longer,^[24]^ while the area of the MTrP decreases after intervention by ischemic compression.^[25]^ Even in the present study, the SCM muscle hardness decreased significantly after SSTR intervention. These results show that applying the release procedure on the soft tissues of the SCM muscle reduced the vertical height and lengthened the sarcomeres and parallel elastic components (including endomysium, perimysium, and epimysium) that cause passive tension in the connective tissues inside the muscle,^[26]^ and this resulted in decreased muscle hardness from reduced resistance by pressure applied to the muscle.^[22,23]^

Sefton et al^[27]^ reported that after applying massage to the neck and shoulders, a decrease in motoneuron pool activity was seen in the H-reflex test on the flexor carpi radialis, which is innervated by the radial nerve that extends from the brachial plexus. In the present study, UT muscle hardness decreased significantly after SSTR intervention. We believe that applying SSTR relaxed the SCM muscle, which reduced the compression on the spinal accessory nerve that passes through the SCM muscle, thereby reducing the motoneuron pool activity of the neuromere that extends from the spinal accessory nerve to the UT muscle, and this had a significant impact on UT muscle hardness.

After SR intervention, UT muscle hardness decreased, thus, because the UT muscle originates from the superior nuchal line and external occipital protuberance, and then passes over the suboccipital muscle, the SR intervention has a direct effect on the UT muscle. This results in a decrease in the muscle hardness.^[28]^

The present study also showed that the PPT of the SCM and UT muscles increased after SSTR intervention; in contrast, after SR was applied, the PPT increased only in the UT muscle. In a comparison of the amount of change between the SSTR and SR interventions, the SSTR intervention showed a significant difference in the PPT of the UT muscle, compared with the SR intervention. Weerapong et al^[29]^ reported that changes in flexibility because of mechanical stimulation on the muscles (release of muscle fibers due to realignment of the muscular structure) was helpful in reducing pain sensation by relaxing the muscles, and that such stimulation increased PPT by blocking pain signals through presynaptic inhibition or by reducing or preventing the pain signal from reaching a conscious level.^[30]^ We believe that mechanical stimulation of the SCM muscle caused a significant increase in PPT of the SCM muscle from reduction of pain sensation and presynaptic inhibition, while relaxation of the SCM muscle had an indirect effect on the significant increase in PPT of the UT muscle, which is innervated by the spinal accessory nerve that passes through the SCM muscle.

After SR intervention, the PPT of the UT muscle increased; this may be because of the direct effect of the SR intervention on the UT muscle, which passes by the upper part of the suboccipital muscle.

Limitations in the present study included the following: because the subjects were smartphone users aged 23∼34 who had latent MTrPs in the UT muscle, the results cannot be generalized; the study did not make a comparison against the SSTR group by applying intervention directly to the UT muscle; only the immediate effects of SSTR and SR interventions on the SCM and suboccipital muscles were examined and the sustained effects of these two interventions on muscle hardness and PPT were not measured; and sonoelastography, one of the most popular approaches, was not used to evaluate muscle hardness. Therefore, additional studies are needed, wherein these limitations are addressed, and the study population includes other people, besides smartphone users, with pain in the UT muscle.

## 5 Conclusions

In the present study, applying SSTR intervention to smartphone users aged 23 to 34 who had latent MTrPs in the UT muscle resulted in decreased muscle hardness and increased PPT in the SCM and UT muscles. When SR intervention was applied, muscle hardness decreased in the UT muscle, whereas PPT increased. When comparing the amount of change before and after the SSTR and SR interventions, significant differences were found for SCM muscle hardness and PPT of the UT muscle. Therefore, we suggest that for reducing pain in the UT muscle, intervention should not only be applied directly to the UT muscle, but SSTR should also be applied to the SCM muscle innervated by the same nerve and SR on the soft tissue surrounding the UT muscle may also be useful.

## Author contributions

**Conceptualization:** Seong-Joong Kim, Jung-hoon Lee.

**Data curation:** Seong-Joong Kim.

**Formal analysis:** Jung-hoon Lee.

**Investigation:** Seong-Joong Kim.

**Methodology:** Seong-Joong Kim, Jung-hoon Lee.

**Project administration:** Seong-Joong Kim, Jung-hoon Lee.

**Software:** Seong-Joong Kim.

**Supervision:** Jung-hoon Lee.

**Writing – original draft:** Seong-Joong Kim, Jung-hoon Lee.

**Writing – review & editing:** Jung-hoon Lee.
